# In vivo tracking of ^14^C thymidine labeled mesenchymal stem cells using ultra-sensitive accelerator mass spectrometry

**DOI:** 10.1038/s41598-020-80416-9

**Published:** 2021-01-14

**Authors:** Min-Seok Oh, Seul-Gi Lee, Gwan-Ho Lee, C-Yoon Kim, Eun-Young Kim, Jong Han Song, Byung-Yong Yu, Hyung Min Chung

**Affiliations:** 1 Department of Stem Cell Biology, School of Medicine, Konkuk University, 120 Neungdong-Ro, Gwangjin-Gu, 05029 Republic of Korea; 2 Advanced Analysis Center, Korea Institute of Science and Technology, Hwarang-ro 14-gil 5, Seongbuk-gu, Seoul, 02792 Republic of Korea; 3 Mirae Cell Bio Co. Ltd, Seoul, 04795 Republic of Korea

**Keywords:** Mass spectrometry, Stem-cell biotechnology, Mesenchymal stem cells

## Abstract

Despite the tremendous advancements made in cell tracking, in vivo imaging and volumetric analysis, it remains difficult to accurately quantify the number of infused cells following stem cell therapy, especially at the single cell level, mainly due to the sensitivity of cells. In this study, we demonstrate the utility of both liquid scintillator counter (LSC) and accelerator mass spectrometry (AMS) in investigating the distribution and quantification of radioisotope labeled adipocyte derived mesenchymal stem cells (AD-MSCs) at the single cell level after intravenous (IV) transplantation. We first show the incorporation of ^14^C-thymidine (5 nCi/ml, 24.2 ng/ml) into AD-MSCs without affecting key biological characteristics. These cells were then utilized to track and quantify the distribution of AD-MSCs delivered through the tail vein by AMS, revealing the number of AD-MSCs existing within different organs per mg and per organ at different time points. Notably, the results show that this highly sensitive approach can quantify one cell per mg which effectively means that AD-MSCs can be detected in various tissues at the single cell level. While the significance of these cells is yet to be elucidated, we show that it is possible to accurately depict the pattern of distribution and quantify AD-MSCs in living tissue. This approach can serve to incrementally build profiles of biodistribution for stem cells such as MSCs which is essential for both research and therapeutic purposes.

## Introduction

The ability to label, track, and image stem cells in real time following transplantation has enabled a wide range of in depth studies that aim to explore and comprehend important biological phenomenon pertinent to regenerative medicine and in particular, stem cell therapy. The use of various types of nanoparticles including quantum dots, superparamagnetic iron oxides (SPIO), silica, and gold in conjunction with fluorescent and photoacoustic imaging techniques as well as other high resolution in vivo imaging methods such as positron emission tomography (PET) and single-photon emission tomography (SPECT), magnetic resonance imaging (MRI), and X-Ray computed microtomography (microCT) has allowed for the long term monitoring of stem cells in model organisms to better understand the behavior of therapeutic cells in both natural and diseased environments^1–8^.

Despite the tremendous advancements made in cell tracking, it remains quite difficult to accurately quantify the number of infused cells within living tissues, especially at the single cell level, mainly due to the sensitivity of cells. And while quantitative PCR is widely used to detect, characterize, and quantify nucleic acids, the method is only relative in the sense that the results will always be proportional to the amount of replicated DNA as opposed to the actual number of active cells^9,10^. Therefore, radioisotope labeling such as carbon-14 thymidine (^14^C-thymidine) which incorporates into new DNA, paired with a liquid scintillator counter (LSC) is commonly employed to measure the radioactivity of ^14^C labeled cells for long-term tracking and quantitative analysis^11,12^. A previous study has shown the tracking of placenta-derived mesenchymal stem cells (PDB-MSCs) in nude mice via ^14^C labeling after IV transplantation, concluding that it is a stable, long term, and quantitative cell tracker^12^. However, LSC is incapable of measuring trace amounts of radioactivity due to its low sensitivity and higher radioisotope concentrations can often affect the functional potential of stem cells such as engraftment, proliferation, secretion, and differentiation. Therefore, a highly sensitive (limits of detection are indicated in terms of attmole (10^–18^) ~ zeptomole (10^–21^) quantities of ^14^C in small samples) method such as accelerator mass spectrometry (AMS) is required to quantify stem cells labeled with low ^14^C thymidine radioactivity without interfering with their normal function^13^. Originally developed for the field of radiocarbon dating in the late 1970s, AMS has proven to be an effective instrument for biomedical and pharmaceutical research when trace quantities of ^14^C need to be quantified in small samples^14,15^. Recently, the tracking of tumor colonization was demonstrated by AMS in a xenograft model which served to quantitatively evaluate metastasis and tumor aggressiveness^16^. While the biological mechanisms and significance behind the distribution of cells throughout time remains largely a mystery due to the limited number of studies, a wider array of data could help to identify general patterns in distribution and localization dependent on cell type and route of delivery.

Herein, we demonstrate using AMS in investigating the distribution and localization of adipocyte derived mesenchymal stem cells (AD-MSCs) at the single cell level after intravenous (IV) transplantation. This study corroborates some of the findings made in previous work but it also reveals the localization of single cells in unexpected organs at early time points. While the significance of these cells is yet to be elucidated, we show that it is possible to accurately depict the pattern of distribution and localization of AD-MSCs in living tissues over time.

## Results

### ***Selection of optimal concentration for ***^***14***^***C-labeling AD-MSCs***

**Figure 1:**
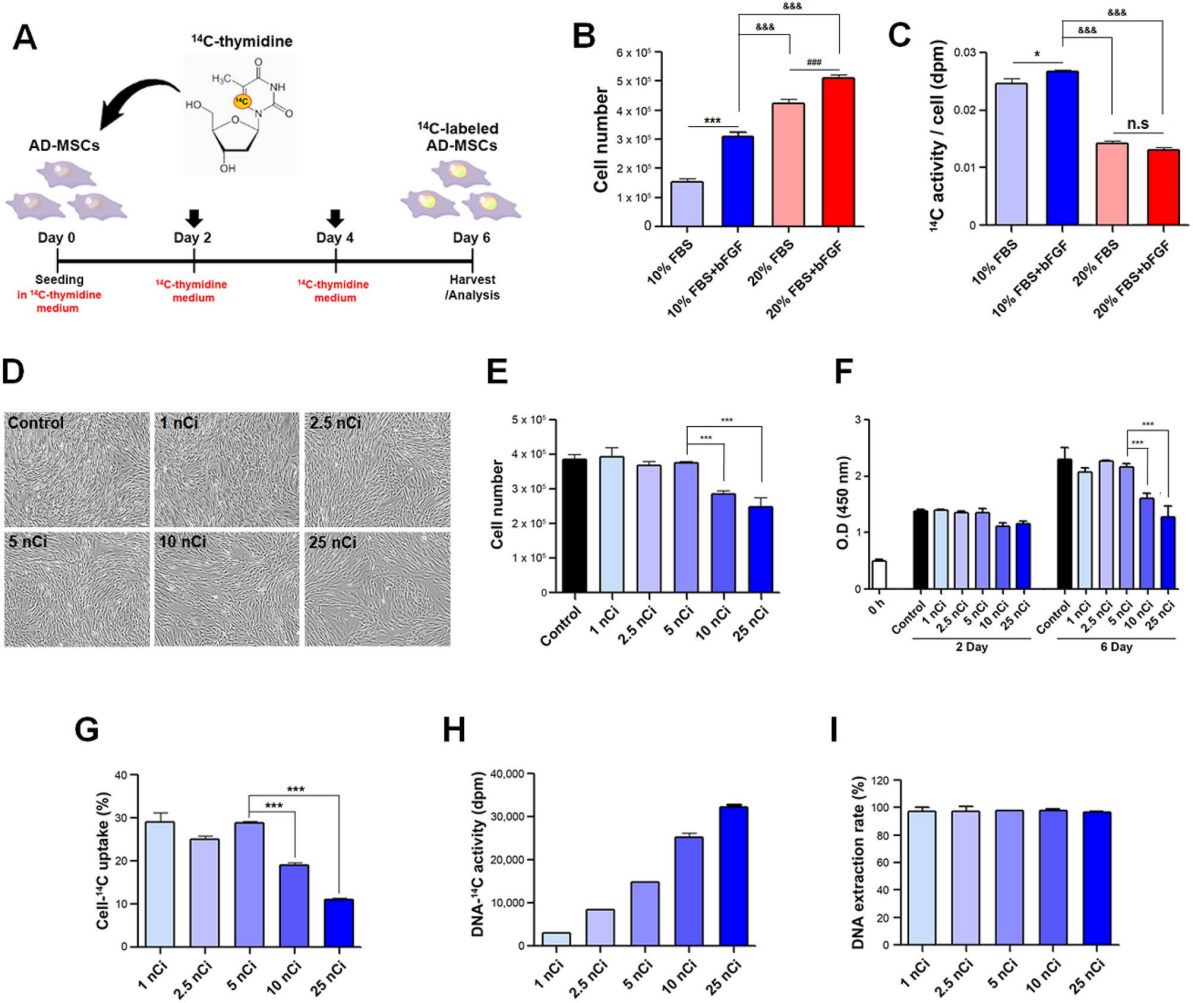
Optimization of ^14^C thymidine concentration for AD-MSCs. (**A**) Method of labeling to prepare ^14^C-labeled AD-MSCs. (**B**) Comparison of cell number by different culture medium condition. (**C**) Comparison of ^14^C activity by different culture medium condition. (**D**) Image for identifying cell number and morphology change by different ^14^C thymidine concentration. (**E**) Cell number comparison by different ^14^C thymidine concentration using hemocytometer. (**F**) Comparison of cell proliferation by different ^14^C thymidine concentration using CCK-8. (**G**) Comparison of uptake rate of AD-MSCs labeled with different concentration. (**H**) Measurement of radioactivity of DNA labeled with different ^14^C thymidine concentration. (**I**) Comparison of extraction rate of DNA. ***p < 0.001 relative to the control or 5 nCi/ml, ^&&&^p < 0.001 relative to the 10% FBS + bFGF group, ^###^p < 0.001 relative to the 20% FBS group. All data are expressed as mean ± SD.

Since ^14^C-thymidine incorporates into the newly replicated DNA when the cell divides, we simply rationalized that AD-MSCs exhibiting a higher proliferation rate would incorporate higher amounts of ^14^C-thymidine during cultivation. Therefore, 2.5 nCi/ml of ^14^C-thymidine was added into various medium conditions (10% FBS, 20% FBS, 10% FBS + bFGF, and 20% FBS + bFGF) to determine the optimal culture group for cell proliferation and consequently, ^14^C-thymidine incorporation (Fig. 1A). Cell counting was performed on day 6 which revealed the proliferative capacity of each group ranking from highest to lowest; 20% FBS + bFGF > 20% FBS > 10% FBS + bFGF > 10% FBS (Fig. 1B). Interestingly and contrary to our expectation, ^14^C activity was significantly higher in the groups containing 10% FBS, especially when supplemented with bFGF, despite the higher proliferation rate observed in those containing 20% FBS (Fig. 1C).

Thus, the 10% FBS + bFGF group was selected to be best suited for both AD-MSC proliferation as well as ^14^C-thymidine incorporation. Next, we sought to determine whether increasing concentrations of ^14^C-thymidine would negatively impact AD-MSC proliferation and morphology by comparing cultivations that received 0 (control), 1, 2.5, 5, 10, or 25 nCi/ml of ^14^C-thymidine (Supplementary Table S1). Based on observation at day 6, it was clear that concentrations of 10 nCi/ml and 25 nCi/ml reduced the proliferation rate of AD-MSCs as more vacant spaces were readily identified in both groups (Fig. 1D). This was further confirmed by cell counting (hemocytometer) and CCK-8 assay which showed a marked decrease in both the total and viable cell population, respectively (Fig. 1E,F). When the level of ^14^C-thymidine uptake for each group was measured as a percentage by LSC, it revealed a decline in uptake efficiency for AD-MSCs that received concentrations of 10 and 25 nCi/ml (Fig. 1G). As expected, higher levels of ^14^C activity was detected as the concentration increased (Fig. 1H) and the ^14^C-thymidine incorporated DNA was able to be extracted for all groups (Fig. 1I). Based on these results, a concentration of 5 nCi/ml was determined to be the optimal concentration for AD-MSCs because ^14^C activity was higher than the 1 and 2.5 nCi/ml groups while the cell proliferation rate and uptake efficiency was maintained, unlike the 10 and 25 nCi/ml groups which exhibited adverse effects.

### Characterization of ^14^C-labeled AD-MSCs

**Figure 2:**
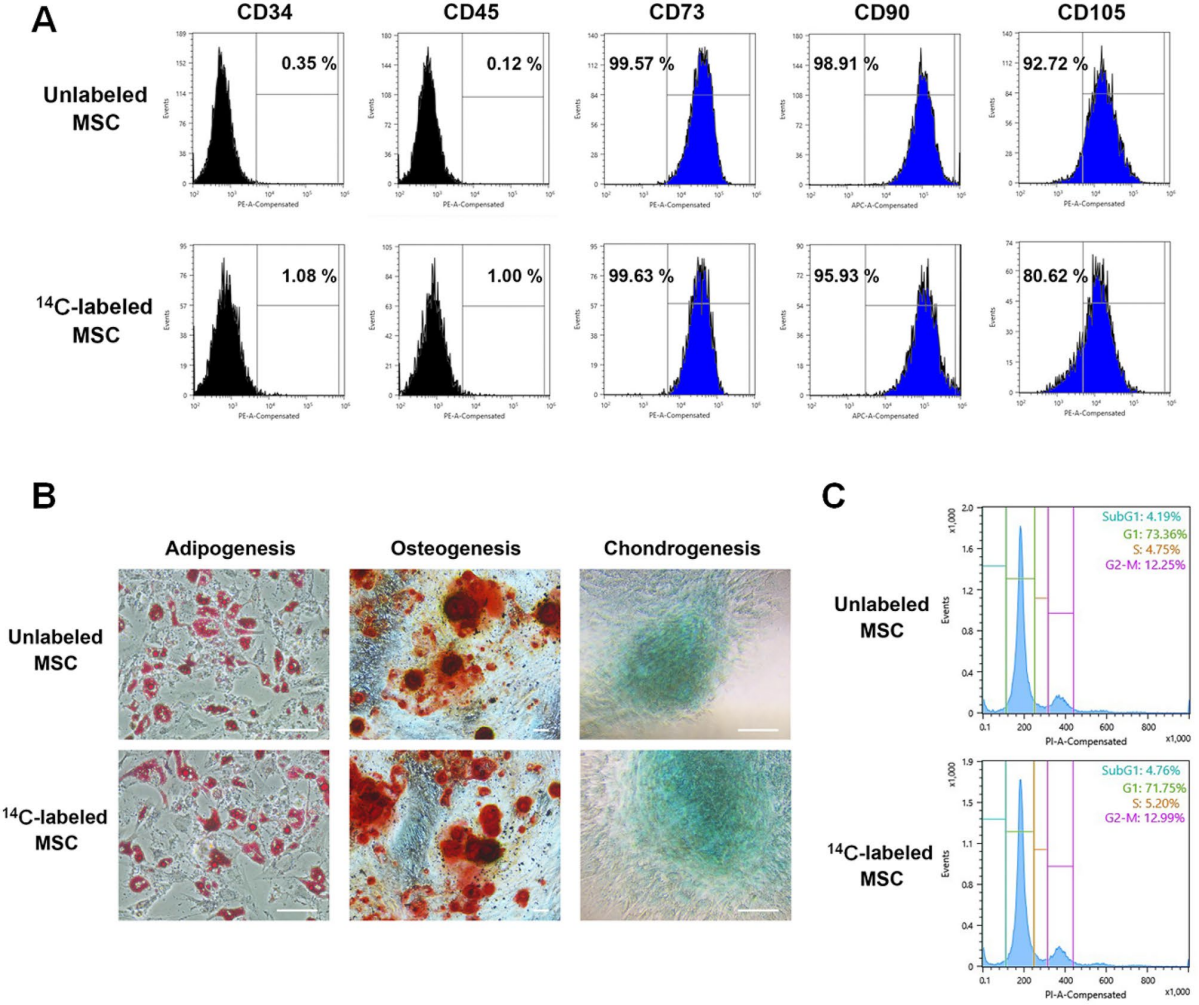
Analysis of biological characteristics of ^14^C-labeled AD-MSCs. (**A**) Confirmation of MSC specific marker expression of unlabeled and ^14^C-labeled AD-MSCs by FACS analysis. (**B**) Investigation of multipotent properties (adipogenesis, osteogenesis, chondrogenesis) of unlabeled and ^14^C-labeled AD-MSCs. (**C**) Comparison of cell cycle analysis of unlabeled and ^14^C-labeled AD-MSCs. Scale bar = 200 µm.

Unlabeled and ^14^C-labeled AD-MSCs were characterized by specific CD marker expression and multi-lineage differentiation to compare surface characters and functional potential. FACS analysis revealed that both populations displayed the hallmark MSC antigen profile of positive CD73, CD90, and CD105 complemented by negative CD34 and CD45 (Fig. 2A). However, a noticeable decrease in CD105 expression was detected as 92.72% of the unlabeled AD-MSCs expressed CD105 which was higher than the 80.62% expressed by the labeled cells. While it remains unclear as to why a difference in marker expression emerged.

The functional characteristics of AD-MSCs and its multipotency were investigated through differentiation. Both groups were subjected to differentiation conditions for adipocytes, osteoblasts, and chondrocytes which were identified by Oil Red O, Alizarin red S, and Alcian blue staining, respectively. Both groups were capable of differentiating into all three cell types, confirming that multipotency was retained in the labeled group (Fig. 2B). In addition, cell cycle analysis was performed to further compare both populations in relation to the different phases of the cell cycle. Both groups shared a very similar profile for the sub-G1, G1, S, and G2-M phases, indicating that cell growth, genome replication, and ultimately the process of mitosis remained unchanged after ^14^C-labeling (Fig. 2C). These results demonstrated that the key characteristics of AD-MSCs in regards to surface markers, multipotency, and cell division were retained in the labeled cells, suggesting that the incorporation of ^14^C-thymidine does not affect the key characteristics of AD-MSCs.

### Pre-treatment process and calibration the LSC and AMS methods

**Figure 3:**
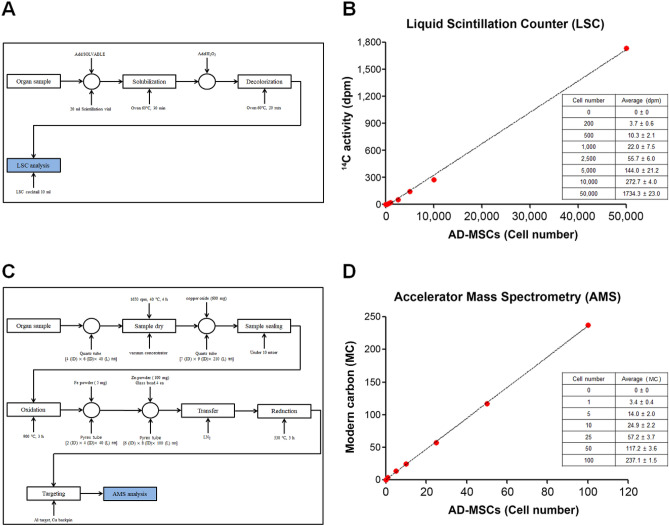
Pretreatment procedure and quantitative curves of LSC and AMS analysis. (**A**) Pretreatment procedure for LSC measurement. (**B**) Calibration curve of LSC using ^14^C activity value (dpm) according to cell number (200, 500, 1,000, 2,500, 5,000, 10,000, and 50,000 cells) (**C**) Pretreatment procedure for AMS measurement. (**D**) Calibration curve of AMS using modern carbon (MC) according to cell number (1, 5, 10, 25, 50, and 100 cells). All data are expressed as mean ± SD.

The detection and quantification of radioactivity by LSC and AMS were compared by first transplanting 50,000 ^14^C-labeled AD-MSCs into a homogenized liver sample before analysis. For LSC, the organ samples were simply solubilized (SOLVABLE, PerkinElmer, Waltham, MA, USA) then decolorized using H_2_O_2_ before sample analysis (Fig. 3A). The LSC calibration curve was prepared using ^14^C activity values (disintegration per minute, dpm) according to the number of labeled AD-MSCs (200, 500, 1000, 2500, 5000, 10,000, and 50,000 cells) and the R^2^ value was 0.9982, indicating linearity (Fig. 3B). Furthermore, to generate a calibration curve for the AMS analysis, 50,000 labeled AD-MSCs were spiked into the homogenized liver sample and diluted to the indicated number of cells (1, 5, 10, 25, 50, and 100 cells). In contrast to LSC, AMS required a comparatively lengthy process that involves the graphitization of organ samples before analysis which is detailed in the Materials and Methods section (Fig. 3C). The diluted ^14^C-labeled AD-MSCs were measured by AMS after pretreatment. The calibration curve of the AMS was prepared using modern carbon (MC, the ratio of ^14^C/^12^C in the reference atmosphere of the 1950s), and a dynamic range of the AMS from 0.1 MC to 150 MC (0.001356 — 2.034 dpm/mgC) according to the number of labeled AD-MSCs. The R^2^ value was 0.9997, indicating linearity (Fig. 3D). When comparing the number of detectable AD-MSCs for each assay, it showed that LSC could not detect less than 200 AD-MSCs. However, a value of 3.4 MC in one cell was obtained from AMS analysis, which was 30 times higher than the limit of detection. These results show that LSC is inadequate in detecting a small number of cells and AMS is required for tracking single cell populations.

### Quantification of ^14^C-labeled AD-MSCs in nude mice by LSC and AMS analyses

In order to investigate the distribution and localization of transplanted AD-MSCs in vivo, nude mice were subjected to IV injection (1 $\times$ 10^6^ cells) and ^14^C radioactivity was measured in the lung, spleen, liver, heart, kidney, and brain by both LSC and AMS at 4 h, 12 h, 24 h, 48 h, and day 7. Using the calibration curve obtained from Fig. 3, the cell concentration and cell amount were calculated from the LSC and AMS measurement of the sample as shown in the following equation. The linear regression equation of calibration curve for quantitation of cell number using LSC was obtained using the cell number and radioactivity, Eq. (1). The cell number was calculated Eq. (2), (3). Where, L_sample_ and L_blank_ are the radioactivity (dpm) of the sample and pre-dose organ, respectively

1 $$Radioactivity\, \left( {dpm} \right) = 0.0349 \times \,\,cell\, number - 21.3$$

2 $$\left( {L_{sample} - L_{blank} } \right) = 0.0349 \, \times \, Cell\, number - 21.3$$

3 $$Cell \,number = \frac{{\left( {L_{sample} - L_{blank} } \right) + 21.3}}{0.0349}$$

The measurement results of AMS were obtained by the ratio of the amount of ^14^C (^14^C_cell_ and ^14^C_organ_) and ^12^C (^12^C_organ_) as shown in the following equation Eq. (4). R_mesu_ and R_blank_ are the value of ^14^C/^12^C of the sample and pre-dose organ, respectively. ^12^C_organ_ is the amounts of carbon (^12^C) contained in the prepared organ. R_sample_ was calculated by subtracting R_mesu_ from R_blank_, Eq. (5).

4 $$R_{mesu} = \frac{{^{14} C_{cell} +^{14} C_{organ} }}{{^{12} C_{organ} }},\quad R_{blank} = \frac{{^{14} C_{organ} }}{{^{12} C_{organ} }}$$

5 $$R_{sample} \left( {MC} \right) = R_{mesu} - R_{blank}$$

The linear regression equation of calibration curve for quantitation of cell number using AMS was obtained using the cell number and modern carbon, Eq. (6). The cell number was calculated Eqs. (7) and (8).

6 $$Modern \,carbon \left( {MC} \right) = 2.356 \times Cell\, number + 0.545$$

7 $$R_{sample} = 2.356 \times Cell\, number + 0.545$$

8 $$Cell\, number = \frac{{R_{sample} - 0.545}}{2.356}$$

Cell concentration (cell number/mg) and cell amount (cell number/organ) can be obtained from cell number, the weight of the sample (W_sample_), and the weight of the organ (W_organ_). Therefore cell concentration and cell amount were calculated, Eq. (9), (10).

9 $${\varvec{Cell}}\,\,\user2{ con}.\,\,\left( {cell\, number{/}mg} \right) = \left( {Cell \,number} \right) \times \frac{1}{{W_{sample} \left( {mg} \right)}}$$

10 $${\varvec{Cell}}\,{\varvec{amount}}\,\user2{ }\left( {cell\, number{/}organ} \right) = \left( {Cell\, number} \right) \times \frac{{W_{organ} \left( {mg{/}organ} \right)}}{{W_{sample} \left( {mg} \right)}}$$

**Figure 4:**
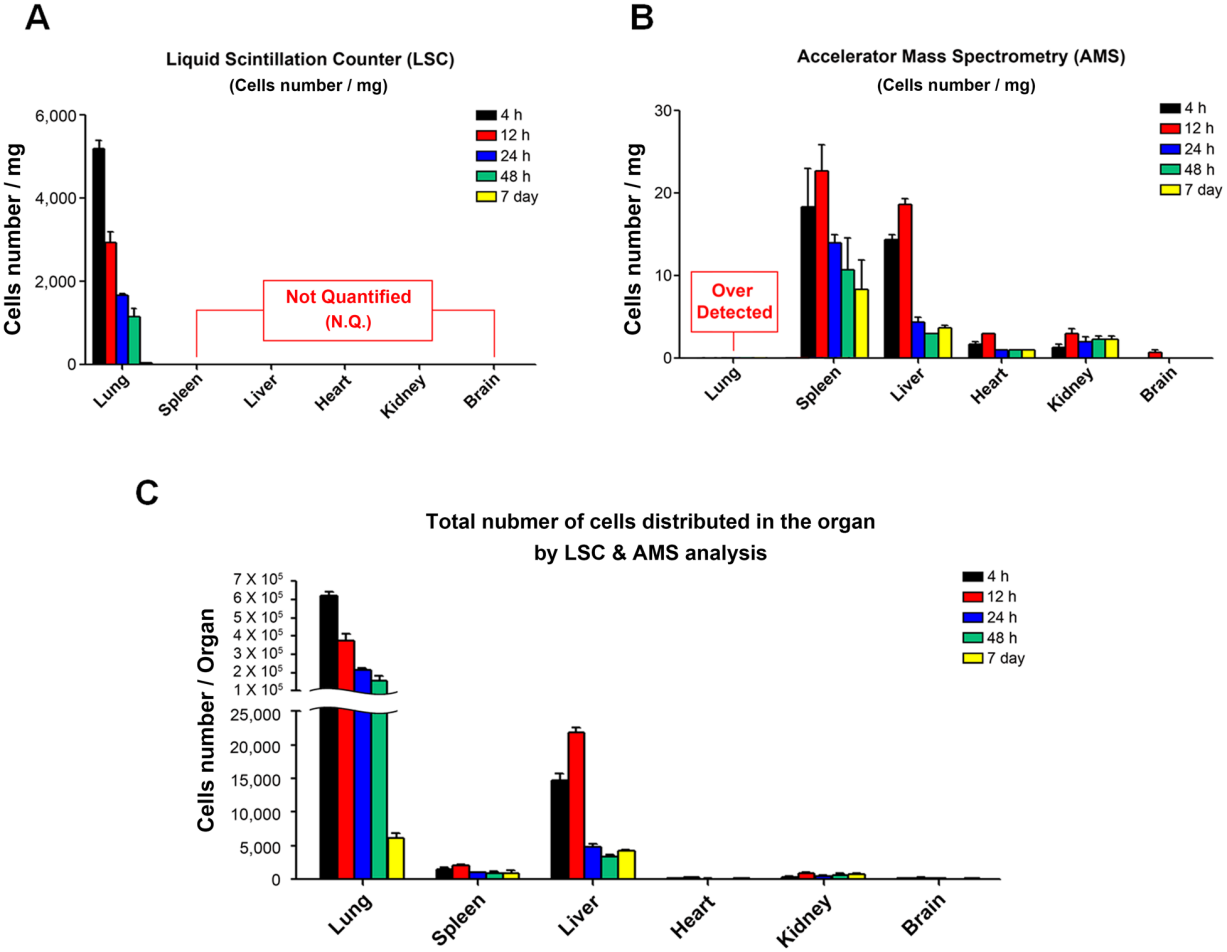
Quntification and distribution results of infused ^14^C-labeled AD-MSCs in nude mice using LSC and AMS analysis. (**A**) The distribution of ^14^C-labeled AD-MSCs after IV injection in nude mice. ^14^C-radioactivity of each organ using LSC. Data were represented as mean cells number/mg. (**B**) Modern carbon (MC) measurement of each organ using AMS. Data were represented as mean cells number/mg. (**C**) Total amount of cells in organs using ^14^C radioactivity value from LSC and AMS analysis. All data are expressed as mean ± SD.

For LSC, AD-MSCs were only detected in the lung with the highest number of cells existing within the organ at 4 h post-transplantation (5,188 ± 365 infused cells/mg). This sharply decreased over time (12 h: 2,930 ± 453, 24 h: 1,673 ± 70, 48 h: 1,161 ± 336) with little to no trace left by 72 h (Fig. 4A). In contrast, AMS was able to detect a small number of cells located in all other tested organs but difficult to quantify larger numbers such as the lung’s case due to over-detection. At 4 h post-transplantation, the number of AD-MSCs per mg located in the spleen and liver was 19 ± 8 and 14 ± 1, respectively. And even lesser amounts or single cells were detected in the heart and kidney (1 ± 0 infused cells number/mg) while no cells were detected in the brain. Interestingly, the number of cells increased at 12 h with exception to the lung which may indicate the occurrence of cell migration from highly populated organs such as the lung to other organs such as the liver and spleen. However, the number of cells was reduced in the liver after 24 h as a fourfold, while other organs (spleen, heart, and kidney) were observed after 12 h as a twofold reduction. Even though the decline continued at 48 h, a small number of residual cells remained within each tissue at day 7 (Fig. 4B). Based on the compiled data of both LSC and AMS, we were able to determine the number of infused cells per organ based on the average mass of each organ to better illustrate AD-MSC localization following IV transplantation (Fig. 4C). The majority of AD-MSCs were present in the lung from 4 h (619,266 ± 36,239) to 48 h (157,448 ± 40,908) and even though the number of active cells continued to decline, ~ 5000 ± AD-MSCs lingered within the organ on day 7. As for the liver, the number of cells significantly increased from 4 h (14,796 ± 1,552) to 12 h (21,941 ± 1,060) before a sharp decline at 24 h (4,761 ± 907). These values were interestingly similar to that of the lung on day 7. The spleen also exhibited a rise in population from 4 h (1,497 ± 590) to 12 h (1,993 ± 454) followed by a decline at 24 h (1,018 ± 156) which plateaued at 48 h (884 ± 593) to day 7 (908 ± 674). This pattern of distribution was similar in the heart and kidney at a much lesser extent (Supplementary Table S2).

### Detection of DiI-labeled AD-MSCs in nude mice

**Figure 5:**
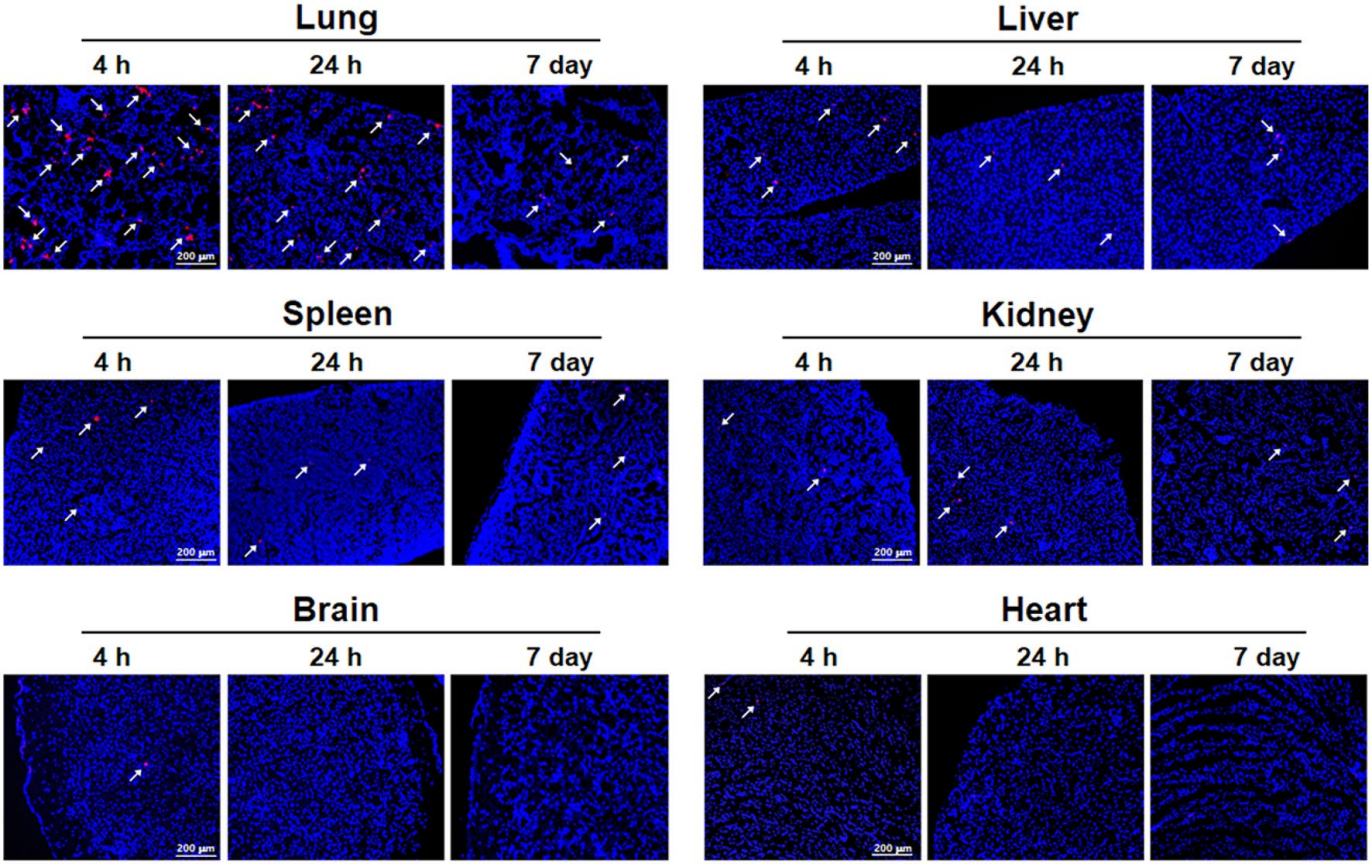
Image of infused AD-MSCs with DiI staining. Distribution of DiI-labeled AD-MSCs were analyzed in lung, liver, spleen, kidney, brain, and heart at 4 h, 24 h, and 7 day after AD-MSCs infused. Scale bar = 200 µm.

To further confirm the successful engraftment of transplanted AD-MSCs and validate the bio-distribution of cells reflected in LSC and AMS, Dil stained AD-MSCs were identified in 5 µm cryosections of each organ at 4 h, 24 h, and 168 h (day 7). And similar to that of ^14^C-labeled AD-MSCs, we found that the majority of cells populated the lung at 4 h followed by the liver and spleen. We also found that the cells began to clear within the first day yet a small number of residual cells remained within the specified organs on day 7. These results complements the tracking of AMS by showing that cells are engrafted visually, although accurate quantification is not possible within each organ (Fig. 5).

## Discussion

This study demonstrates the successful incorporation of ^14^C-thymidine (5 nCi/ml) into AD-MSCs without affecting key biological characteristics pertinent to morphology, CD marker expression, differentiation, and cell cycle. These cells were then utilized to track and quantify the distribution of AD-MSCs delivered through an intravenous route (tail vein) by AMS, revealing the number of AD-MSCs existing within different organs per mg and per organ at different time points. Notably, the results show that this highly sensitive approach can quantify one cell per mg which effectively means that AD-MSCs can be detected in various tissues at the single cell level.

Even though the history of AMS is extensive such as Geoscience, Archaeology, Environmental Science, and Biomedical Science, its adaptation to the field of regenerative medicine is fairly recent as only a few studies documented its usage in the tracking and quantification of distributed stem cells in vivo. Previously reported radioisotope labeling methods such as ^51^Cr, ^124^I, and ^99m^TC often require highly radioactivity for imaging purposes; 18.5 MBq (500 µCi) for ^51^Cr, 3.7 MBq/0.1 ml (100 µCi/0.1 ml) for ^124^I-FIAU, and 3.7 MBq (100 µCi) for ^64^Cu-PISM^17,18^. Furthermore, studies that employ a β-counter, often used concentrations of 1 µCi of ^3^H-thymidine or ^14^C-thymidine which has also been estimated to cause cell damage^12,19–21^. In this study, we show that far lower concentrations can be used for the long term tracking of AD-MSCs following transplantation due to long half-life of ^14^C (half-life = 5,370 years). Since stem cells exhibit plasticity unlike somatic cells, it is very likely that they are more prone to change upon interference thereby a highly sensitive approach is advantageous to ensure that its character is not disrupted before administration.

Another important factor for cell distribution is the route of delivery. Although our study is limited to IV, it is well known that other delivery routes, whether systematic (IV) or local (direct injection) can change the distributive outcome. For example in IV and intra-atrial (IA), cells are able to be distributed throughout several organs with most of the cells accumulating within the lung, liver, and spleen as shown in this study which is also consistent with previous reports^9,22,23^. In contrast, direct injection (DI) can target a specific tissue but prohibits interactions between MSCs and secondary signaling systems^24^. And because there is no consensus on the optimal delivery route for MSCs at this time, the approach used in this study can help to elucidate the distribution and localization of stem cells following transplantation through a selected route in order to further determine effective routes for various cell types. In sum, radioisotope labeling paired with AMS can be a very useful tool in tracking and quantifying AD-MSCs at the single cell level after transplantation. Future studies should focus on applying a similar approach to other stem cell variants of both multipotent and pluripotent origin to incrementally build profiles of biodistribution which can serve to accelerate our understanding of stem cell behavior in vivo especially in light of the increasing attention and clinical studies surrounding stem cell therapeutics.

## Materials and methods

### Cell culture and labeling of ^14^C-tymidine

Human adipose-derived mesenchymal stem cells (hAD-MSCs; ATCC, Manassas, VA, USA) were cultured in Dulbecco’s Modified Eagle Media (DMEM; Gibco BRL, Grand Island, NY) supplemented with 10% fetal bovine serum (FBS; Thermo Fisher Scientific, Waltham, MA, USA), 1% GlutaMax (Gibco), 1% Non-Essential Amion Acids Solution (MEM NEAA; Gibco), 1% penicillin–streptomycin (P/S; Thermo Fisher Scientific) and 10 ng/ml basic fibroblast growth factor (bFGF). Cells were maintained at 37 °C in a humidified atmosphere containing 5% CO_2_. For labeling of [2-^14^C]-thymidine (Moravek Biochemicals, Brea, CA, USA) in the AD-MSC, AD-MSCs were seeded at 7.8 $\times$ 10^5^ cells in 100 mm culture dish (46 cells/mm^2^) with culture medium containing ^14^C-tymidine at the indicated concentration (1, 2.5, 5, 10, and 25 nCi/ml; 4.8, 12.1, 24.2, 48.4, and 121.1 ng/ml). After 2 days and 4 days, the medium was replaced with culture medium containing ^14^C-tymidine and cells were detached using 0.25% trypsin–EDTA (Gibco) in 6 days.

### Cell proliferation assay

Cell viability was examined via a Cell Counting Kit-8 (CCK-8; Dojindo Molecular Technologies, Kumamoto, Japan) according to the manufacture’s protocol. Cells were seeded into 96-well plates at a density of 2,000 cells/well in a final volume of 100 µl and grown under culture medium containing indicated concentration of ^14^C-thymidine. The cells were cultured for 2 day and 6 day; then, 10 µl CCK-8 reagent was added to each well and the cells were incubated at 37 °C for 4 h. Cell viability was calculated by measuring the absorbance at 450 nm with a microplate reader.

### DNA extraction

DNA was harvested from ^14^C labeled AD-MSCs grown in culture. Cultured ^14^C AD-MSCs were washed in PBS prior to 2 ~ 3 $\times$ 10^5^ cells was digested with Proteinase K, RNase, and AL buffer using commercially available silica-based columns (DNeasy Blood & Tissue Kit, Qiagen, Hilden, Germany). Concentration of isolated DNA was quantified using a NanoDrop one Microvolume UV–Vis Spectrophotometer (Thermo Fisher Scientific, Waltham, MA, USA).

### Analysis of liquid scintillation counter (LSC)

The activity of the incorporated ^14^C-thymidine into the DNA of AD-MSCs was measured using Tri-Carb 4910TR LSC instrument (Perkin Elmer, Shelton, CT, USA). ^14^C labeled AD-MSC and extracted DNA in PBS (DNA concentration, 100 μl) of aliquot from each sample were added into 10 ml of LSC cocktail (Perkin Elmer, Shelton, CT, USA). Each radioactivity in mixed solution was measured through LSC for 30 min.

### Characterization of 14C-labeled adipose-derived mesenchymal stem cells

Characterization of AD-MSC was conducted according to our previously described method^25^. Cells were detached by 0.25% trypsin–EDTA at ~ 90% passage, detached cells were resuspended in FACS buffer (PBS solution including 0.5% bovine albumin (BSA) and 2 mm EDTA) and filtered using a premoistened a 40-µm cell strainer. Cells were then labeled using each antibody of MSC surface markers according to manufacturer’s instructions. Types of antibody are as follows. Fluorochrome-conjugated antibodies for CD73-PE, CD90-APC, and CD105-PE (BD Biosciences, Bedford, MA, USA), along with a negative marker CD34 and CD45 conjugated to PE (BD Biosciences) were used. Also, corresponding IgG controls were prepared equally, and 30,000 labeled cells were acquired and analyzed using Becton Dickinson FACS Calibur (BD Biosciences).

### Assessment of multilineage differentiation

Multilineage differentiation of AD-MSC was conducted according to our previously described method^25^. For the induction of osteoblasts, adipocytes, and chondroblasts, commercially available kits were used (Thermo Fisher Scientific, Waltham, MA, USA). Cells under differentiation conditions were maintained in 4-well plates or 12-well plates. Osteogenesis was incubated for 21 days, and adipogenic and chondrogenic lineage was induced for 14 days. All experimental procedures were performed according to the manufacturer’s instructions. To evaluate each differentiation process, appropriate staining was performed: Alizarin Red S to identify calcium deposits, Oil Red O to detect intracellular lipid droplets, and Alcian blue to confirm the formation of proteoglycans. Images were analyzed using and inverted microscope.

### Intravenous injection of ^14^C-labeled AD-MSCs in nude mice

BALB/c nude mice (6 weeks old, male, weight 19–22 g) were supplied by Orient Bio (Seongnam, Republic of Korea). All experimental procedures were performed according to the guidelines and with the approval of the Institutional Animal Care and Use Committee (IACUC) of the Korea Institute of Science and Technology (IACUC number: KIST-2019–002). Housed at the Integrated Animal Center of the Korea Institute of Science and Technology (KIST) maintained on a 12 h interval day and night cycle. Mice were allowed free access to water and feed. In order to confirm the distribution of ^14^C-labeled AD-MSCs after the transplantation of cells into mice, five different groups of mice were prepared for assessment at 4 h, 12 h, 24 h, 48 h and 7 days after intravenous (IV) injection (n = 3). Then, close to 1 $\times$ 10^6^ of ^14^C-labeled AD-MSCs suspended in 150 μl of PBS were injected into each mouse via the tail vein. After the injection of the ^14^C-labeled AD-MSCs, the mice were sacrificed with CO_2_ and organs (lung, spleen, liver, heart, kidney, brain) were extracted. In order to prevent ^14^C contamination between organs during separation, each part was separated a set of forceps that underwent frequent cleaning them with PBS. The extracted organs were washed with PBS and then frozen in a cryo-tube. Samples were stored in a -80 °C until further processing. All animal experiments were approved by an ethics committee organized in KIST.

### Graphitization of samples and accelerator mass spectrometry (AMS) measurement

In order to homogenize collected organ were washed 2 times in PBS prior to organ homogenization using a BeadBug microtube homogenizer (Benchmark scientific, Edison, NJ, USA) in TEN buffer. After homogenization, about ~ 25 μl of each organ sample were dried using vacuum concentrator (LABCONCO, Kansas City, Mo, USA) and copper oxide (600 mg) were added to the sample tube followed by placing into a quartz tube for oxidation. Briefly, dried sample were sealed by the propane-oxygen flame under vacuum and oxidized at 900 °C for 3 h in muffle furnace (JISICO, Seoul, Republic of Korea). The oxidized gases (importantly isotopologues of gaseous carbon dioxide) were transferred to an in situ reduction tube via cryogenic trapping by transfer system manufactured at KIST; the non-condensable gases at liquid nitrogen temperature (-196 °C) were removed under vacuum and the carbon dioxide(s) reacted at 530 °C for 6 h to form graphite over the iron catalyst^26^. The graphite generated on the iron powder was mixed using a metal rod and then pressed using a KIST pressing tool to generate the target for AMS measurement. The KIST AMS is a 6MV Tandentron (HVEE, High Voltage Engineering Europa, BV, The Netherlands) with a terminal voltage set to 3 MV to accelerate carbon atomic ions(C^-^);the system background was ^14^C/^12^C =  ~ 5 $\times$ 10^–16^ (as determined using pyrolytic graphite sheet). SRM 4990C (1.3407 Modern Carbon; Oxalic acid II) standard sample provided by National Institute of Standards and Technology (NIST) were prepared and measured for the calibration of AMS samples. C3 (1.2941 Modern carbon, cellulose) and C8 (0.1503 Modern carbon, oxalic acid) as reference materials (obtained from IAEA) were also prepared for the confirming sample treatment and AMS measurement process. In general at least 50,000 “counts” were acquired to obtain under under-1% imprecision. The measured ^14^C/^12^C ratios of each sample were normalized to the Oxalic acid II results and the final Modern carbon values were calculated.

### Detection of DiI labeled AD-MSCs in nude mice

The organs were dehydrate in 4% PFA with a graded sucrose series then embedded in OCT compound to obtain 5 µm cryo section. This was used to identify AD-MSCs with DiI staining.

### Statistical analysis

Statistical analyses were performed using the by GraphPad Prism software (La Jolla, CA, USA; Version 5). Data are presented as mean ± SD, and the statistical significance of the experimental results were calculated using one-way ANOVA. A value of p < 0.05 was considered statistically significant.

## Supplementary information

Supplementary Information 1.
