# Supplementary material for: In vivo tracking of 14C thymidine labeled mesenchymal stem cells using ultra-sensitive accelerator mass spectrometry
Source: Sci Rep. 2021 Jan 14;11:1360. doi: 10.1038/s41598-020-80416-9 (PMC7809063; doi:10.1038/s41598-020-80416-9)
Supplement: Supplementary file 1 — Supplementary Information 1. [file 41598_2020_80416_MOESM1_ESM.docx]

***In Vivo* Tracking of ^14^C Thymidine Labeled Mesenchymal Stem Cells Using Ultra-Sensitive Accelerator Mass Spectrometry**

Min-Seok Oh^1†^, Seul-Gi Lee^1†^, Gwan-Ho Lee^2^, C-Yoon Kim^1^, Eun-Young Kim^3^, Jong Han Song^2^, Byung-Yong Yu^2*^, Hyung Min Chung^1,3*^

^1^ Department of Stem Cell Biology, School of Medicine, Konkuk University, 120 Neungdong-Ro, Gwangjin-Gu, Seoul Republic of Korea, 05029

^2^ Advanced Analysis Center, Korea Institute of Science and Technology, Hwarang-ro 14-gil 5, Seongbuk-gu, Seoul, Republic of Korea, 02792

^3^ Mirae Cell Bio Co. Ltd, Seoul, Republic of Korea, 04795

^†,*^ These authors contributed equally to this work as first and corresponding authors, respectively.

*** Corresponding Author**

: Hyung Min Chung, PhD

Department of Stem Cell Biology, School of Medicine, Konkuk University, 120 Neungdong-Ro, Gwangjin-Gu, Seoul, Republic of Korea, 05029

Tel, +82 2 2049 6028; E-mail, hmchung@kku.ac.kr

: Byung-Yong Yu, PhD

Advanced Analysis Center, Korea Institute of Science and Technology, Hwarang-ro 14-gil 5, Seongbuk-gu, Seoul, Republic of Korea, 02792

Tel, +82 2 958 5099; E-mail, yu2997@kist.re.kr

**Supplementary Tables**

**Supplementary Table S1** Comparison of cell number and ^14^C radioactivity according to the dose of ^14^C thymidine.

| ^14^C-thymidine dose (nCi/ml) | Seeded cells in 6 well plate (number) | Harvested cells after 6 days (number) | ^14^C radioactivity  (dpm) | ^14^C radioactivity (dpm/cell) |
| --- | --- | --- | --- | --- |
| 1 | 50,000 | 393,000 ± 29,000 | 3,226.7 ± 250.2 | 0.008 ± 0.001 |
| 2.5 |  | 368,000 ± 12,500 | 8,682.7 ± 260.0 | 0.024 ± 0.001 |
| 5 |  | 377,000 ± 4,200 | 15,277.3 ± 187.7 | 0.041 ± 0.001 |
| 10 |  | 286,000 ± 9,600 | 25848.0 ± 739.4 | 0.090 ± 0.003 |
| 25 |  | 248,000 ± 28,000 | 33,262.7 ± 834.3 | 0.136 ± 0.019 |

**Supplementary Table S2** Tracking of cell distribution in each organ over time using LSC and AMS analysis. Quantification of cell number per organ by LSC and AMS analysis. Results are expressed in mean percentage ± standard deviation, with MPI values in brackets.

| LSC | | | | | | |
| --- | --- | --- | --- | --- | --- | --- |
| Time (h) | Cell number/Organ | | | | | |
|  | Lung | Spleen | Liver | Heart | Kidney | Brain |
| 4 | 619,266 ± 36,239 | ^*^N.Q. | N.Q. | N.Q. | N.Q. | N.Q. |
| 12 | 374,990 ± 60,657 | N.Q. | N.Q. | N.Q. | N.Q. | N.Q. |
| 24 | 213,917 ± 18,636 | N.Q. | N.Q. | N.Q. | N.Q. | N.Q. |
| 48 | 157,448 ± 40,908 | N.Q. | N.Q. | N.Q. | N.Q. | N.Q. |
| 168 (7day) | 6,179 ± 1,295 | N.Q. | N.Q. | N.Q. | N.Q. | N.Q. |

^*^Not quantification

| AMS | | | | | | |
| --- | --- | --- | --- | --- | --- | --- |
| Time (h) | Cell number/Organ | | | | | |
|  | Lung | Spleen | Liver | Heart | Kidney | Brain |
| 4 | - | 1,497 ± 590 | 14,796 ± 1,552 | 140 ± 18 | 380 ± 93 | 108 ± 33 |
| 12 | - | 1,993 ± 454 | 21,941 ± 1,060 | 264 ± 28 | 828 ± 280 | 222 ± 58 |
| 24 | - | 1,018 ± 156 | 4,761 ± 907 | 86 ± 25 | 533 ± 175 | 108 ± 37 |
| 48 | - | 884 ± 593 | 3,390 ± 459 | 82 ± 1 | 635 ± 332 | 90 ± 9 |
| 168 (7day) | - | 908 ± 674 | 4,265 ± 170 | 135 ± 28 | 778 ± 237 | 120 ± 11 |
